# Supplementary material for: Novel Amperometric Biosensor Based on Tyrosinase/Chitosan Nanoparticles for Sensitive and Interference-Free Detection of Total Catecholamine
Source: Biosensors (Basel). 2022 Jul 12;12(7):519. doi: 10.3390/bios12070519 (PMC9313403; doi:10.3390/bios12070519)
Supplement: Supplementary file 1 [file biosensors-12-00519-s001.zip › biosensors-1804255-supplementary.pdf]

biosensors

MDPI

Supplementary Information

Novel Amperometric Biosensor Based on Tyrosinase/Chitosan Nanoparticles for Sensitive and Interference-Free Detection of Total Catecholamine

Table S1. Electrochemical parameters related to GPH/SPE and ChitNPs/GPH/SPE modiefied electrode: electroactive area (A_{EA}), roughness factor (ρ) and heterogenous electron transfer rate costant (K_0). Experimental condition: 1.1 mM $Fe(CN)_6^{4-}$ in 0.1 M KCl.

	A_{EA} / cm²	ρ	K_0 10^{-3} / cm s^{-1}
GPH/SPE	0.130 ± 0.002	1.15	3.2 ± 0.6
ChitNPs/GPH/SPE	0.170 ± 0.003	1.38	3.6 ± 0.8

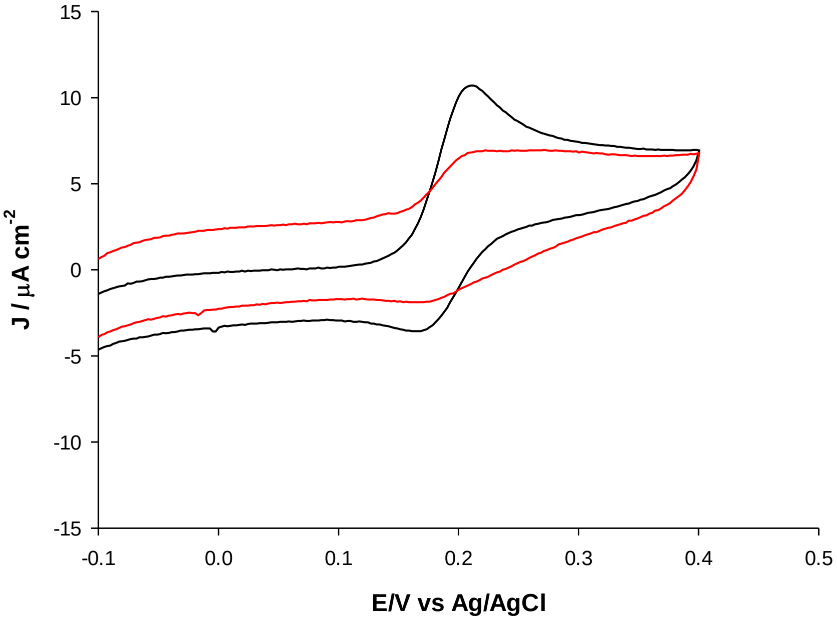

Figure S1. CVs of 50 µM Dopamine in PBS 0.1 M (pH 7.2; KCl 0.1 M), on a ChitNPs/GPH/SPE (black line) and Chit/GPH/SPE (red line).

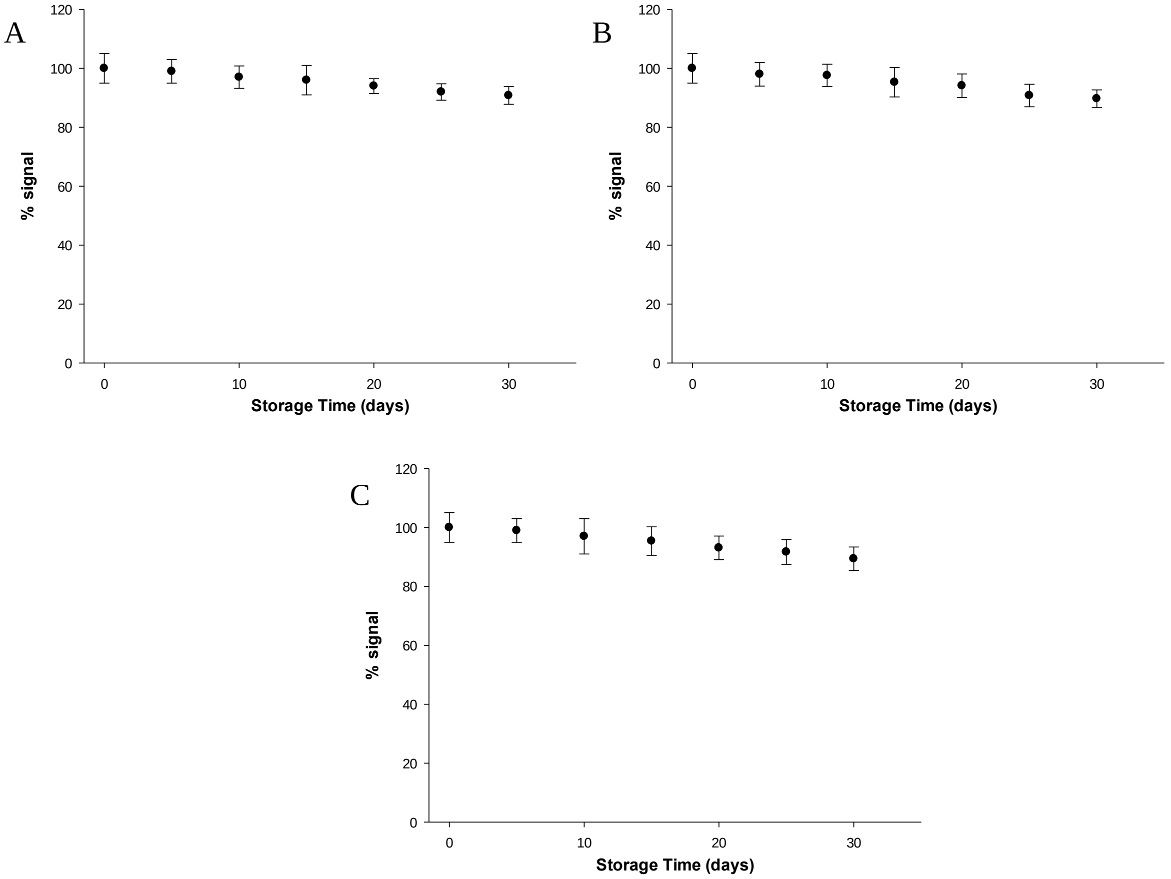

Figure S2. Stability measurements over a period of 30 days in presence of 10 µM of DA (Panel A), EP (Panel B) and NEP (Panel C) in PBS 0.1 M (pH 7.2; KCl 0.1 M).

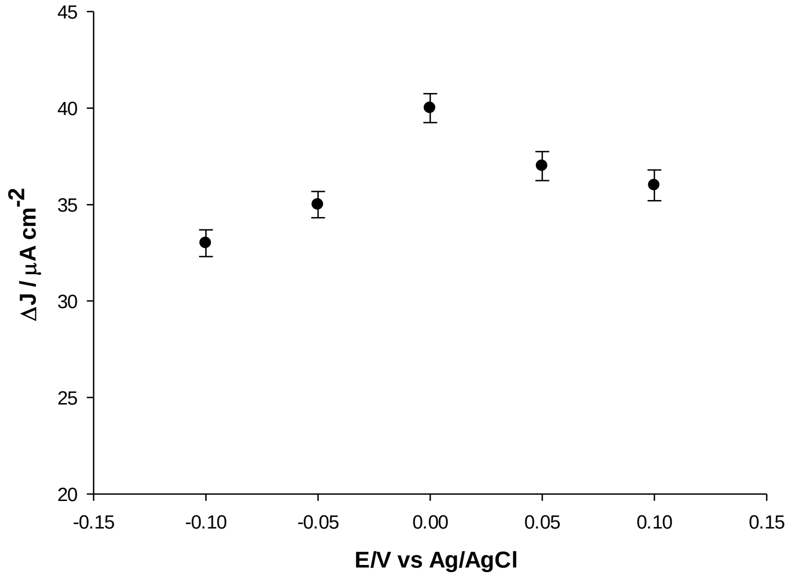

Figure S3. Optimization of applied potential with Tyr/EDC-NHS/ChitNPs/GPH/SPE in 50 mM dopamine in 0.1 M PBS (pH 7.2; KCl 0.1 M).
